# Supplementary material for: China’s plastic import ban increases prospects of environmental impact mitigation of plastic waste trade flow worldwide
Source: Nat Commun. 2021 Jan 18;12:425. doi: 10.1038/s41467-020-20741-9 (PMC7813828; doi:10.1038/s41467-020-20741-9)
Supplement: Supplementary file 1 — Supplementary Information [file 41467_2020_20741_MOESM1_ESM.pdf]

China's plastic import ban increases prospects of environmental impact mitigation of plastic waste trade flow worldwide

Zongguo Wen [a,*], Yiling Xie [a], Muhan Chen, Christian Dinga Doh [a]

[a] State Key Joint Laboratory of Environment Simulation and Pollution Control (SKLESPC), School of Environment, Tsinghua University, Beijing, 100084, China

Supplementary Information

Table

Supplementary Table 1. The trade flows of global plastic waste market from Comtrade in Baseline Scenario (Unit: t). (Corresponding to Figure 1.)

Importer \ Exporter		China	HK	Japan	USA	Europe 7							Southeast Asia 5					Rep. of Korea	Mexico
						Germany	UK	Belgium	Spain	Italy	France	Netherlands	Thailand	Indonesia	Viet Nam	Malaysia	Philippines		
China		-	3184175.95	886254.83	923632.15	646162.82	250897.51	156735.41	94609.23	65512.82	94057.74	75735.84	179284.22	135518.06	144236.28	112363.75	78434.85	169200.67	176279.98
HK		1596.15	-	565702.33	617155.36	218161.42	314871.38	102921.61	64400.91	28945.86	74226.33	83014.33	42709.20	6031.62	2564.16	29802.23	7324.76	23130.29	80515.24
Japan		166.73	291.84	-	1022.78	41.42	131.32	22.30	66.07	19.04	18.29	19.88	407.93	37.62	342.91	129.98	187.41	485.02	32.08
USA		11205.60	253.44	1464.73	-	17088.81	865.05	7240.96	574.81	9101.83	1937.62	740.24	3190.17	428.80	83.97	145.35	0.00	60.80	196139.03
Europe 7	Germany	65.40	16.13	52.89	444.71	-	21886.17	12690.01	4538.03	17219.74	52637.83	56769.02	14.43	6.95	5.74	10.43	0.00	5.68	208.77
	UK	983.76	70.95	3.64	376.45	11768.82	-	4701.95	2055.84	1589.38	8337.56	30454.74	5.87	34.91	12.47	16.69	3.03	2.43	1301.93
	Belgium	68.78	17.66	40.93	733.36	19693.76	4938.12	-	3316.50	3481.38	44212.85	64652.89	9.24	2.31	169.71	6.11	13.34	11.09	332.89
	Spain	6.55	0.00	51.92	1208.10	7097.77	2397.20	1171.51	-	7844.42	40966.99	5722.73	1.44	10.24	0.00	0.00	4.79	0.00	199.87
	Italy	49.56	5.94	24.52	490.28	18133.00	5536.21	13113.49	18300.14	-	61549.33	7332.30	16.56	87.61	0.00	5.62	47.83	2.97	83.74
	France	59.16	2.49	14.00	65.29	25031.83	12855.58	20799.92	3338.85	6663.28	-	13044.71	0.25	62.69	4.51	5.99	4.25	0.06	2025.04
	Netherlands	155.42	4.82	22.34	427.49	100453.37	31389.83	51391.32	10490.47	2212.32	29464.68	-	2.61	10.39	0.34	16.42	150.10	7.17	17.57
Southeast Asia 5	Thailand	2403.39	2313.43	11954.30	7640.08	531.64	2848.23	1933.75	3984.13	38.33	593.53	21.78	-	130.74	604.51	4904.34	163.48	59.50	316.76
	Indonesia	237.68	254.63	525.01	40721.46	511.80	17226.87	2761.35	1336.03	110.90	110.13	663.37	5.76	-	197.80	8126.37	232.05	644.61	10571.53
	Viet Nam	125.06	87620.23	39209.54	28688.45	12279.63	23608.13	10232.66	13219.85	1779.51	1688.46	6128.33	10938.67	351.58	-	3450.21	512.63	10399.08	650.35
	Malaysia	2642.70	4773.11	19747.90	25603.06	27949.84	34249.27	6581.52	13421.61	648.60	1315.38	690.01	3325.32	1598.88	914.94	-	871.62	7481.40	2343.64
	Philippines	371.20	1467.83	538.64	1141.61	10.44	527.03	155.12	121.03	0.63	60.82	0.30	93.09	42.65	101.56	232.55	-	50.14	83.44
Rep. of Korea		1233.97	4298.79	40892.33	7713.79	1024.99	3178.26	2373.64	155.22	27.21	599.12	645.82	1127.42	2687.52	6399.48	2880.50	8021.28	-	1927.74
Mexico		57.54	2.51	2.54	22452.24	238.84	14.64	165.43	53.72	25.29	9.46	4.97	0.05	0.00	0.00	0.67	0.00	0.74	-

Supplementary Table 2. The trade flows of global plastic waste market from Comtrade in 2018 Scenario (Unit: t). (Corresponding to Figure 2.) *Data with the orange background were estimated on the basis of monthly data, mirror data, and others.

Importer \ Exporter		China	HK	Japan	USA	Europe 7							Southeast Asia 5					Rep. of Korea	Mexico
						Germany	UK	Belgium	Spain	Italy	France	Netherlands	Thailand	Indonesia	Viet Nam	Malaysia	Philippines		
China		-	49858.28	45969.68	0.00	13486.19	13860.30	4686.96	17215.99	5552.81	2974.70	1076.81	26634.67	61220.42	33174.34	11514.47	19673.12	10617.24	3070.73
HK		15635.38	-	54299.49	0.00	72976.55	39783.61	23433.78	14234.68	1199.04	12626.63	8584.80	3549.82	640.67	2091.39	1827.17	1121.18	267.82	25910.36
Japan		1436.40	394.66	-	663.82	1136.18	41.33	20.00	171.89	0.11	50.01	0.02	851.08	24.96	231.58	2017.63	125.63	952.92	31.34
USA		11625.58	682.90	9172.71	-	12199.23	3437.91	7720.00	2265.87	4388.82	1441.74	354.55	7830.10	6106.69	2490.94	2376.22	3247.19	0.57	194974.64
Europe 7	Germany	284.72	0.00	3729.65	3458.58	-	22685.73	33015.68	12053.26	26719.02	36140.90	72150.70	48.75	23.40	0.00	1.07	0.00	0.00	21.65
	UK	1425.05	78.48	66.83	553.82	27033.61	-	5748.43	496.84	2277.67	18424.20	41558.27	1063.41	622.74	0.00	0.00	16.35	0.00	480.31

	**Belgium**	131.76	0.00	158.87	2832.39	25040.25	20699.46	-	1356.10	714.12	35793.13	53501.88	0.00	1600.07	31.01	4.80	0.00	285.91	89.36
	**Spain**	105.51	0.00	2211.04	5225.30	8903.91	13821.58	1993.50	-	17913.80	80663.01	2309.63	359.62	206.08	975.82	0.00	407.85	0.06	382.72
	**Italy**	0.00	0.00	558.22	447.99	19344.40	8164.86	12596.71	4980.87	-	114408.97	13081.59	252.15	1143.66	0.00	0.00	6.21	0.00	656.25
	**France**	101.18	0.00	209.49	0.00	25220.81	17587.38	32820.64	8475.83	8348.71	-	13569.36	0.00	20.00	0.00	60.25	0.00	0.00	2563.84
	**Netherlands**	209.78	96.09	1611.11	1865.27	121246.39	50841.96	154628.30	2640.54	700.89	36694.62	-	0.00	20.00	0.46	0.00	17.10	9.68	138.08
**Southeast Asia 5**	**Thailand**	19835.96	115001.23	187826.61	106929.19	2045.72	14379.92	331.73	13460.66	3628.90	676.75	53.02	-	2250.85	68.08	10132.64	836.44	6864.43	8165.70
	**Indonesia**	1747.41	4661.64	20449.54	27385.17	64458.92	71929.27	13835.20	4030.47	213.90	3293.98	24910.96	306.81	-	1.64	7218.53	162.50	600.15	1571.94
	**Viet Nam**	218.60	31750.91	123255.16	74433.85	56781.45	23422.60	27582.36	25049.40	5501.89	7511.82	14289.04	11516.16	3722.25	-	1528.75	4332.32	26129.18	1772.18
	**Malaysia**	7100.45	70634.43	220398.93	207044.51	131453.22	102087.61	33066.65	28748.06	12909.95	17681.83	8102.70	3132.81	5330.31	0.00	-	744.29	11111.56	20815.48
	**Philippines**	101.36	3382.13	11485.18	6530.44	433.64	1347.14	120.54	238.24	19.90	195.55	0.10	718.34	1151.01	0.00	45.01	-	8755.86	706.68
**Rep. of Korea**		2902.68	1196.48	101321.37	35510.22	7074.30	3967.92	7105.79	692.33	853.54	832.91	2562.90	27735.08	1663.89	15053.58	322.02	6843.16	-	2836.16
**Mexico**		151.51	0.00	0.00	46201.22	888.50	0.00	727.95	0.99	909.31	25.68	87.24	231.76	0.00	1.47	0.00	5.20	20.00	-

Supplementary Table 3. The data of ED and TFCR of exports. (Corresponding to Figure 3.)

**Countries and regions**	HK	Japan	USA	Germany	UK	Belgium	Spain	Italy	France	Netherlands	Thailand	Indonesia	Viet Nam	Malaysia	Philippines	Rep. of Korea	Mexico
**Total export flow of Baseline (t)**	3290609.84	1657497.37	1979033.84	1363297.61	785685.89	437973.55	261225.50	219580.12	469302.57	413033.14	252411.03	148832.98	162611.90	176027.96	107699.82	212952.97	488776.15
**ED**	96.77%	87.60%	77.86%	63.40%	72.01%	59.29%	60.87%	43.02%	35.86%	38.44%	87.95%	95.11%	90.28%	80.76%	79.63%	90.32%	52.54%
**TFCR of exports**	-0.9125	-0.3918	-0.5409	-0.2339	-0.1873	0.2119	-0.3364	-0.1024	-0.0572	-0.1635	-0.2448	-0.3381	-0.6375	-0.7380	-0.3954	-0.6831	-0.3679

Supplementary Table 4. The proportion of types of plastics and three common treatment options for plastic waste.

Country or region	Proportion of plastic types (PE: PS: PVC: PET: PP: other)	Proportion of plastic waste treatments (Landfill or mismanagement: Incineration: Recycling)	
		Baseline	2018
China mainland	0.170: 0.019: 0.145: 0.233: 0.140: 0.293 [1]	0.695: 0.116: 0.189 [1]	0.676: 0.124: 0.200 [1]
Hong Kong, China	0.462: 0.048: 0.041: 0.183: 0.266: 0 [2]	0.550: 0.255: 0.195 [3]	0.550: 0.255: 0.195 [3]
Japan	0.231: 0.073: 0.158: 0.037: 0.221: 0.280 [4]	0.080: 0.670: 0.250 [5]	0.060: 0.660: 0.280 [6]
USA	0.386: 0.083: 0.038: 0.118: 0.185: 0.190 [7]	0.754: 0.155: 0.091 [8]	0.758: 0.158: 0.084 [9]
Germany	0.298: 0.066: 0.102: 0.074: 0.193: 0.267 [10]	0.005: 0.605: 0.390 [11]	0.010: 0.610: 0.380 [12]
The UK	0.298: 0.066: 0.102: 0.074: 0.193: 0.267 [10]	0.290: 0.390: 0.320 [11]	0.220: 0.470: 0.310 [12]
Belgium	0.298: 0.066: 0.102: 0.074: 0.193: 0.267 [10]	0.020: 0.650: 0.330 [11]	0.020: 0.650: 0.330 [12]
Spain	0.298: 0.066: 0.102: 0.074: 0.193: 0.267 [10]	0.480: 0.150: 0.370 [11]	0.390: 0.190: 0.420 [12]
Italy	0.298: 0.066: 0.102: 0.074: 0.193: 0.267 [10]	0.370: 0.340: 0.290 [11]	0.370: 0.320: 0.310 [12]
France	0.298: 0.066: 0.102: 0.074: 0.193: 0.267 [10]	0.340: 0.430: 0.230 [11]	0.340: 0.420: 0.240 [12]
Netherlands	0.298: 0.066: 0.102: 0.074: 0.193: 0.267 [10]	0.010: 0.660: 0.330 [11]	0.000: 0.670: 0.330 [12]
Thailand	0.462: 0.048: 0.041: 0.183: 0.266: 0 [2]	0.754: 0.030: 0.216 [13,14]	0.754: 0.030: 0.216 [13,14]
Indonesia	0.462: 0.048: 0.041: 0.183: 0.266: 0 [2]	0.827: 0.021: 0.152 [13,14]	0.827: 0.021: 0.152 [13,14]
Viet Nam	0.462: 0.048: 0.041: 0.183: 0.266: 0 [2]	0.879: 0.015: 0.106 [13,14]	0.879: 0.015: 0.106 [13,14]
Malaysia	0.462: 0.048: 0.041: 0.183: 0.266: 0 [2]	0.570: 0.052: 0.378 [13,14]	0.570: 0.052: 0.378 [13,14]
Philippines	0.462: 0.048: 0.041: 0.183: 0.266: 0 [2]	0.827: 0.021: 0.152 [13,14]	0.827: 0.021: 0.152 [13,14]
Rep. of Korea	0.462: 0.048: 0.041: 0.183: 0.266: 0 [2]	0.241: 0.379: 0.380 [15]	0.241: 0.379: 0.380 [15]
Mexico	0.462: 0.048: 0.041: 0.183: 0.266: 0 [2]	0.950: 0.000: 0.050 [16]	0.950: 0.000: 0.050 [16]
Other Countries	0.462: 0.048: 0.041: 0.183: 0.266: 0 [2]	0.550: 0.255: 0.195 [3]	0.550: 0.255: 0.195 [3]

Supplementary Table 5. EIT and Eco-costs of the China ban for 2018 Scenario. (Corresponding to Figure 5.)

Indicator	Unit		Eco-costs value (Euro/kg)	EIT				Eco-costs (Euro)			
				Total	(DM)	(Exp)	(tran)	Total	(DM)	(Exp)	(tran)
**global warming (GW)**	kg CO2 eq	(unspecified)	1.16E-01	**1.3420E+08**	**2.0220E+09**	**-6.2736E+08**	**-1.2605E+09**	1.5567E+07	2.3456E+08	-7.2774E+07	-1.4621E+08
**fine particulate matter formation (FPMF)**	kg PM2.5 eq	high. pop.	6.69E+01	**-5.2880E+06**	**-3.5206E+06**	**2.9319E+06**	**-4.6993E+06**	-3.5350E+08	-2.3535E+08	1.9600E+08	-3.1415E+08
		(unspecified)	3.50E+01					-1.8508E+08	-1.2322E+08	1.0262E+08	-1.6448E+08
		low. pop.	3.19E+00					-1.6869E+07	-1.1231E+07	9.3527E+06	-1.4991E+07
**freshwater ecotoxicity (FWE)**	kg 1,4DCB-eq to fresh water	(unspecified)	3.37E-02	**-5.6537E+07**	**8.3346E+08**	**-8.8261E+08**	**-7.3850E+06**	-1.9048E+06	2.8080E+07	-2.9736E+07	-2.4881E+05
		ocean	2.29E-05					-1.2964E+03	1.9111E+04	-2.0238E+04	-1.6933E+02
**human carcinogenic toxicity (HCT)**	kg 1,4DCB-eq to urban air	high. pop.	2.50E-01	**-2.4664E+07**	**-5.7302E+07**	**3.8488E+07**	**-5.8496E+06**	-6.1719E+06	-1.4339E+07	9.6313E+06	-1.4638E+06
		(unspecified)	1.67E-01					-4.1070E+06	-9.5420E+06	6.4091E+06	-9.7408E+05
		low. pop.	8.25E-02					-2.0354E+06	-4.7288E+06	3.1762E+06	-4.8273E+05
**water consumption (WC)**	m3	(unspecified)	6.60E-01	**-3.2900E+09**	**-4.0813E+09**	**9.8747E+08**	**-1.9613E+08**	-2.1714E+09	-2.6937E+09	6.5173E+08	-1.2945E+08
**Eco-costs**	Euro	(Max)						-2.5174E+09	-2.6807E+09	7.5485E+08	-5.9152E+08
								**-2.3469E+09**	**-2.5638E+09**	**6.5825E+08**	**-4.4136E+08**
		(Min)						-2.1747E+09	-2.4751E+09	5.9147E+08	-2.9114E+08

Supplementary Table 6. The unit values on GW of countries with different incineration rates and per capita GDP. (The data of Per capita GDP are from the World Bank- https://data.worldbank.org.cn/)

Countries	China	HK, China	Japan	USA	Germany	UK	Belgium	Spain	Italy	France	Netherlands	Thailand	Indonesia	Viet Nam	Malaysia	Philippines	Rep. of Korea	Mexico
**Incineration rate in 2018**	0.124	0.255	0.660	0.158	0.610	0.470	0.650	0.190	0.320	0.420	0.670	0.030	0.021	0.015	0.052	0.021	0.379	0.000
**Per capita GDP in 2018 (Unchanged dollar in 2010)**	7,755.00	38,784.80	48,919.60	54,541.70	47,501.80	42,986.00	46,683.20	33,146.40	35,391.70	43,663.60	55,022.90	6,361.60	4,284.70	1,964.50	12,109.50	3,022.00	26,761.90	10,385.30
**Unit impact values of domestic management in 2018 on GW (kg CO_2 eq / 1 kg plastic waste)**	-0.04922	0.21615	0.88237	0.27757	0.56220	0.44472	0.86006	-0.31868	0.15707	0.60528	0.76390	-0.14075	-0.05690	0.00404	-0.35410	-0.05690	0.19529	0.05311

Supplementary Table 7. Sensitivity analysis of some parameters of LCA in 2018 Scenario.

Parameter		EIT					EIT(DM)					EIT(Exp)					EIT(tran)				
		GW (kg CO_2 eq)	FPMF (kg PM2.5 eq)	FEW (kg 1,4-DCB)	HCT (kg 1,4-DCB)	WC (m^3)	GW (kg CO_2 eq)	FPMF (kg PM2.5 eq)	FEW (kg 1,4-DCB)	HCT (kg 1,4-DCB)	WC (m^3)	GW (kg CO_2 eq)	FPMF (kg PM2.5 eq)	FEW (kg 1,4-DCB)	HCT (kg 1,4-DCB)	WC (m^3)	GW (kg CO_2 eq)	FPMF (kg PM2.5 eq)	FEW (kg 1,4-DCB)	HCT (kg 1,4-DCB)	WC (m^3)
Road transportation distance	50 km	2.18E+08	-5.25E+06	-5.49E+07	-1.95E+07	-3.09E+09	1.98E+09	-3.64E+06	8.33E+08	-5.73E+07	-4.08E+09	-5.64E+08	3.01E+06	-8.82E+08	4.11E+07	1.09E+09	-1.20E+09	-4.62E+06	-6.44E+06	-3.25E+06	-9.81E+07
	100 km	1.34E+08	-5.29E+06	-5.65E+07	-2.47E+07	-3.29E+09	2.02E+09	-3.52E+06	8.33E+08	-5.73E+07	-4.08E+09	-6.27E+08	2.93E+06	-8.83E+08	3.85E+07	9.87E+08	-1.26E+09	-4.70E+06	-7.39E+06	-5.85E+06	-1.96E+08
	200 km	-3.28E+07	-5.36E+06	-5.98E+07	-3.50E+07	-3.68E+09	2.11E+09	-3.29E+06	8.34E+08	-5.72E+07	-4.08E+09	-7.54E+08	2.78E+06	-8.84E+08	3.33E+07	7.91E+08	-1.39E+09	-4.85E+06	-9.27E+06	-1.10E+07	-3.92E+08
Unit avoided electricity /1 kg	0.85 kWh	1.43E+08	-5.33E+06	-5.58E+07	-2.35E+07	-3.04E+09	2.11E+09	-3.34E+06	8.34E+08	-5.51E+07	-3.81E+09	-7.04E+08	2.71E+06	-8.83E+08	3.75E+07	9.68E+08	-1.26E+09	-4.70E+06	-7.39E+06	-5.85E+06	-1.96E+08
	0.9 kWh	1.34E+08	-5.29E+06	-5.65E+07	-2.47E+07	-3.29E+09	2.02E+09	-3.52E+06	8.33E+08	-5.73E+07	-4.08E+09	-6.27E+08	2.93E+06	-8.83E+08	3.85E+07	9.87E+08	-1.26E+09	-4.70E+06	-7.39E+06	-5.85E+06	-1.96E+08
	0.95 kWh	1.26E+08	-5.24E+06	-5.73E+07	-2.59E+07	-3.54E+09	1.94E+09	-3.70E+06	8.33E+08	-5.95E+07	-4.35E+09	-5.50E+08	3.16E+06	-8.82E+08	3.94E+07	1.01E+09	-1.26E+09	-4.70E+06	-7.39E+06	-5.85E+06	-1.96E+08

Unit avoided plastic production /1 kg	0.86 kg	1.67E+08	-5.26E+06	-5.64E+07	-2.38E+07	-3.28E+09	2.19E+09	-3.38E+06	8.34E+08	-5.30E+07	-4.00E+09	-7.62E+08	2.82E+06	-8.83E+08	3.51E+07	9.19E+08	-1.26E+09	-4.70E+06	-7.39E+06	-5.85E+06	-1.96E+08
	0.91 kg	1.34E+08	-5.29E+06	-5.65E+07	-2.47E+07	-3.29E+09	2.02E+09	-3.52E+06	8.33E+08	-5.73E+07	-4.08E+09	-6.27E+08	2.93E+06	-8.83E+08	3.85E+07	9.87E+08	-1.26E+09	-4.70E+06	-7.39E+06	-5.85E+06	-1.96E+08
	0.96 kg	1.02E+08	-5.31E+06	-5.67E+07	-2.55E+07	-3.30E+09	1.85E+09	-3.66E+06	8.33E+08	-6.16E+07	-4.16E+09	-4.92E+08	3.05E+06	-8.82E+08	4.19E+07	1.06E+09	-1.26E+09	-4.70E+06	-7.39E+06	-5.85E+06	-1.96E+08
Unit electricity consumption /1 kg	0.55 kWh	1.51E+08	-5.20E+06	-5.69E+07	-2.51E+07	-3.44E+09	1.96E+09	-3.67E+06	8.33E+08	-5.89E+07	-4.26E+09	-5.45E+08	3.16E+06	-8.82E+08	3.96E+07	1.02E+09	-1.26E+09	-4.70E+06	-7.39E+06	-5.85E+06	-1.96E+08
	0.6 kWh	1.34E+08	-5.29E+06	-5.65E+07	-2.47E+07	-3.29E+09	2.02E+09	-3.52E+06	8.33E+08	-5.73E+07	-4.08E+09	-6.27E+08	2.93E+06	-8.83E+08	3.85E+07	9.87E+08	-1.26E+09	-4.70E+06	-7.39E+06	-5.85E+06	-1.96E+08
	0.65 kWh	1.17E+08	-5.37E+06	-5.62E+07	-2.42E+07	-3.14E+09	2.09E+09	-3.37E+06	8.34E+08	-5.57E+07	-3.90E+09	-7.09E+08	2.70E+06	-8.83E+08	3.74E+07	9.54E+08	-1.26E+09	-4.70E+06	-7.39E+06	-5.85E+06	-1.96E+08
$\textbf{Sensitivity coefficient} = \left(\frac{\Delta EIT_i}{EIT_i}\right) \Big/ \left(\frac{\Delta \text{Parameter}_i}{\text{Parameter}_i}\right)$																					
Road transportation distance		-1.24	0.01	0.06	0.42	0.12	0.04	-0.07	0.00	0.00	0.00	0.20	-0.05	0.00	-0.14	-0.20	0.10	0.03	0.26	0.89	1.00
Unit avoided electricity		-1.15	-0.15	0.23	0.89	1.36	-0.76	0.93	-0.02	0.68	1.18	-2.21	1.38	0.00	0.45	0.36	0.00	0.00	0.00	0.00	0.00
Unit avoided plastic production		-4.43	0.09	0.05	0.64	0.07	-1.51	0.72	-0.02	1.36	0.36	-3.92	0.70	-0.02	1.62	1.25	0.00	0.00	0.00	0.00	0.00
Unit electricity consumption		-1.54	0.20	-0.08	-0.23	-0.55	0.38	-0.50	0.01	-0.33	-0.54	1.57	-0.95	0.00	-0.34	-0.41	0.00	0.00	0.00	0.00	0.00

Supplementary Table 8. Data of environmental impacts used in LCA of plastic waste trade flow change.

Data of	Selected datasets (“products” or “processes” in SimaPro)	Source database
Transportation	**Road**<br>Transport, lorry >32t, EURO5/RER U;<br>Transport, lorry 16-32t, EURO5/RER U;<br>Transport, lorry 3.5-16t, fleet average/RER U;<br>Transport, refuse truck, diesel powered/tkm/RNA	Ecoinvent unit processes<br><br><br>USLCI
	**Ocean**<br>Transport, ocean freighter, average fuel mix/US	USLCI
Waste treatment	**Landfill**<br>Waste plastic, mixture | treatment of, sanitary landfill | APOS, U;<br>Waste polyethylene | treatment of, sanitary landfill | APOS, U;<br>Waste polyethylene terephthalate | treatment of, sanitary landfill | APOS, U;<br>Waste polypropylene | treatment of, sanitary landfill | APOS, U;<br>Waste polystyrene | treatment of, sanitary landfill | APOS, U;<br>Waste polyvinylchloride | treatment of, sanitary landfill | APOS, U;<br>**Incineration**<br>Treatment of waste plastic, mixture, municipal incineration | APOS, U;<br>Treatment of waste polyethylene, municipal incineration | APOS, U;<br>Treatment of waste polyethylene terephthalate , municipal incineration with fly ash extraction | APOS, U;<br>Treatment of waste polypropylene, municipal incineration | APOS, U;<br>Treatment of waste polystyrene, municipal incineration | APOS, U;<br>Treatment of waste polyvinylchloride, municipal	Ecoinvent – allocation at point of substitution – unit

	incineration | APOS, U.<br>(each process ahs two versions: Europe without Switzerland and RoW)	
Electricity	Electricity, medium voltage, at grid/CN U (China) and national grid data of Japan (JP), the U.S. (US), Germany (DE), the UK (GB), Belgium (BE), Spain (ES), Italy (IT), France (FR), and Netherland (NL)	Ecoinvent unit processes
Plastic production (used to model avoided products)	**PE**<br>Polyethylene, HDPE, granulate, at plant/RER U ;<br>Polyethylene, LDPE, granulate, at plant/RER U ;<br>Polyethylene, LLDPE, granulate, at plant/RER U;<br>**PS**<br>Polystyrene, expandable, at plant/RER U;<br>**PVC**<br>Polyvinylchloride, at regional storage/RER U;<br>**PET**<br>Polyethylene terephthalate, granulate, amorphous, at plant/RER U;<br>Polyethylene terephthalate, granulate, bottle grade, at plant/RER U;<br>**PP**<br>Polypropylene, granulate, at plant/RER U;<br>**Other**<br>Mean value of the data above	Ecoinvent unit processes

Supplementary Table 9. Comparison among environmental impacts of changes in waste flows and treatment structure in different scenarios.

Scenario / Value / Indicator	2018	Exports Scenarios				Recycling rate Scenarios			Combination Scenarios	
		**Zero exports**	**Half of exports**	**Half of exports of developed countries**	**Half of exports of developing countries**	**20% increase recycling rate**	**50% increase recycling rate**	**100% increase recycling rate**	**Half of exports +20% increase recycling rate**	**Half of exports +50% increase recycling rate**
GW (kg CO_2 eq)	1.34E+08	1.06E+09	5.97E+08	7.60E+08	-2.94E+07	1.49E+07	-6.03E+07	-4.57E+07	4.94E+08	5.42E+08
FPMF (kg PM2.5 eq)	-5.29E+06	-1.04E+07	-7.85E+06	-7.55E+06	-5.58E+06	-5.55E+06	-5.88E+06	-6.32E+06	-8.18E+06	-8.54E+06
FWE (kg 1,4-DCB eq)	-5.65E+07	-8.86E+07	-7.26E+07	-8.07E+07	-4.84E+07	-6.04E+07	-5.84E+07	-4.39E+07	-7.33E+07	-5.91E+07
HCT (kg 1,4-DCB eq)	-2.47E+07	-2.53E+07	-2.50E+07	-2.67E+07	-2.29E+07	-2.57E+07	-2.46E+07	-1.92E+07	-2.54E+07	-2.10E+07
WC (m_3)	-3.29E+09	-5.76E+09	-4.53E+09	-4.53E+09	-3.29E+09	-2.97E+09	-2.59E+09	-1.99E+09	-4.22E+09	-3.90E+09
Eco-costs (Euro/year)	-2.35E+09	-4.05E+09	-3.20E+09	-3.17E+09	-2.37E+09	-2.16E+09	-1.93E+09	-1.54E+09	-3.02E+09	-2.82E+09
Values of 2018 scaled to 1:										

GW	1	7.90	4.45	5.67	-0.22	0.11	-0.45	-0.34	3.68	4.04
FPMF	-1	-1.97	-1.48	-1.43	-1.06	-1.05	-1.11	-1.19	-1.55	-1.61
FWE	-1	-1.57	-1.28	-1.43	-0.86	-1.07	-1.03	-0.78	-1.30	-1.05
HCT	-1	-1.03	-1.01	-1.08	-0.93	-1.04	-1.00	-0.78	-1.03	-0.85
WC	-1	-1.75	-1.38	-1.38	-1.00	-0.90	-0.79	-0.61	-1.28	-1.19
**Eco-costs**	-1	-1.73	-1.36	-1.35	-1.01	-0.92	-0.82	-0.66	-1.29	-1.20

Supplementary Table 10. The trade flows of global plastic waste market from Comtrade in 2019 Scenario (Unit: t). *Data with the orange background were estimated on the basis of mirror data and blank table indicate missing data.

Exporter / ***Importer***		**China**	**HK**	**Japan**	**USA**	**Europe 7**							**Southeast Asia 5**					**Rep. of Korea**	**Mexico**
						**Germany**	**UK**	**Belgium**	**Spain**	**Italy**	**France**	**Netherlands**	**Thailand**	**Indonesia**	**Viet Nam**	**Malaysia**	**Philippines**		
**China**		-	271.45	19202.23	16910.39	2632.46	9413.02	2669.71		5910.63								1853.62	
**HK**			-	57348.57	72743.29	93110.15	62231.39	39360.66		808.12								702.53	
**Japan**			71.74	-	585.37	278.31	44.33											398.65	
**USA**			43.79	15727.87	-	14377.44	1022.80	3422.62		1623.67								378.41	
**Europe 7**	**Germany**		141.42	2639.76	1137.18	-	31764.61	28042.34		18561.86								2.00	
	**UK**		2.68	68.52	816.68	33162.28	-	4188.88		122.24									
	**Belgium**		46.63	358.55	2297.52	26367.45	19509.02	-		718.62									
	**Spain**		0.00	2385.16	4397.18	5139.39	28967.13	1283.21	-	8005.43								0.00	
	**Italy**			168.66	510.91	17784.37	12614.03	7901.47		-								2.98	
	**France**		0.00	143.57	142.70	20055.86	22105.80	42605.79		8605.05	-							0.00	
	**Netherlands**		0.00	490.93	246.79	142980.36	54251.69	97427.62		587.35		-						0.00	
**Southeast Asia 5**	**Thailand**		91625.38	102332.80	27218.89	1550.30	1194.41	128.74		373.51			-					3115.51	
	**Indonesia**		1365.52	16929.25	24338.91	34337.97	22537.93	14817.02		24.71				-				701.42	
	**Viet Nam**		80438.07	116616.64	21620.17	14892.05	152.82	16252.57		526.83					-			13546.90	
	**Malaysia**		51127.54	261743.04	60625.26	182486.96	0.00	62533.05		11542.51						-		8857.19	
	**Philippines**		1627.76	13907.65	7888.33	339.19	0.00	22.96		0.00							-	517.12	
**Rep. of Korea**			498.73	88878.90	30980.46	6932.30	4450.72	6107.97		46.07								-	
**Mexico**			0.00	0.00	36943.18	668.44	0.00	439.68		1279.82								0.00	

Supplementary Table 11. The trade flows of global plastic waste market from Comtrade in 2017 Scenario (Unit: t).

Importer \ ***Exporter***		**China**	**HK**	**Japan**	**USA**	**Europe 7**							**Southeast Asia 5**					**Rep. of Korea**	**Mexico**
						**Germany**	**UK**	**Belgium**	**Spain**	**Italy**	**France**	**Netherlands**	**Thailand**	**Indonesia**	**Viet Nam**	**Malaysia**	**Philippines**		
**China**		-	1710971.30	749380.74	557875.21	345836.66	164174.98	113768.77	115911.32	40115.32	77750.26	69736.50	333515.11	173938.16	265923.12	115844.79	55826.87	119574.90	95952.68
**HK**		297.61	-	274759.41	373147.81	99193.96	77543.60	31566.27	46982.88	2081.25	36971.76	18918.54	41311.27	5431.13	5237.53	14838.75	4541.55	7028.83	55338.94
**Japan**		491.19	165.70	-	1260.55	32.45	21.70	378.05	159.43	0.00	0.04	2.87	344.68	2.90	2232.21	38.20	947.08	921.00	2.94
**USA**		10073.82	148.97	3523.87	-	16109.13	920.38	6190.06	300.82	6436.50	1631.93	291.36	2388.78	2731.83	4.71	567.01	0.01	233.46	190040.84
**Europe 7**	**Germany**	168.57	0.00	14.62	1043.22	-	23256.09	30690.85	9399.43	28798.71	35090.53	76820.36	0.64	0.02	0.00	20.46	0.00	0.00	59.30
	**UK**	1254.35	0.00	65.70	339.61	24373.77	-	4233.22	1217.28	3296.77	6810.53	59056.86	0.00	10.16	4.90	0.00	0.00	0.00	364.12
	**Belgium**	54.40	23.74	45.05	1254.25	21017.35	8786.10	-	1222.33	1687.07	28001.62	47440.94	0.00	140.00	0.00	0.00	0.00	0.00	157.15
	**Spain**	23.80	0.00	1106.62	8593.95	8003.49	1967.38	1772.73	-	13583.31	66445.41	2650.57	0.00	0.00	0.00	25.94	0.00	0.00	65.89
	**Italy**	26.65	0.00	40.00	15.37	20707.45	6036.46	11534.47	27518.18	-	79222.28	11059.11	0.00	360.00	0.00	0.00	0.00	0.00	92.38
	**France**	0.00	0.00	0.00	59.01	22556.00	14125.24	34068.43	9514.41	7342.75	-	11353.74	0.00	0.39	3.82	0.00	0.00	0.00	80.65
	**Netherlands**	164.61	0.00	0.00	1570.67	141680.89	45146.57	97022.05	3093.74	1941.17	38013.87	-	0.00	0.00	0.00	0.00	0.00	0.00	0.00
**Southeast Asia 5**	**Thailand**	2467.13	10919.68	58160.48	32862.36	2702.27	2420.70	3462.11	4187.33	417.06	270.90	290.49	-	590.45	429.75	6813.29	214.14	604.24	386.95
	**Indonesia**	722.80	474.01	2699.51	29174.87	564.62	38592.05	4935.94	790.49	0.00	439.91	1387.40	16.87	-	58.67	1986.25	68.94	401.52	3402.77
	**Viet Nam**	294.73	21595.30	126219.22	137193.47	69242.47	54312.66	44336.58	23601.20	5959.17	6200.51	9291.73	18951.28	1183.68	-	1316.39	104.60	50787.06	2548.31
	**Malaysia**	2166.88	32885.50	75435.09	121139.66	75242.09	91468.71	18051.47	22213.22	4369.80	15455.43	2948.30	534.39	2192.18	157.01	-	1028.92	12984.90	5233.71
	**Philippines**	0.00	4895.09	2138.91	3635.06	176.00	1418.58	76.98	195.36	0.00	0.00	0.96	236.83	147.42	46.10	41.96	-	4397.46	283.09
**Rep. of Korea**		91.82	760.55	33258.10	9139.37	842.63	4593.84	2964.16	266.56	39.64	115.55	1048.54	107.41	1583.94	17737.25	300.38	2428.00	-	655.18
**Mexico**		134.73	0.00	0.00	47175.43	1333.57	0.00	862.57	244.37	293.64	27.74	1.36	0.00	0.00	0.00	0.00	0.00	6.22	-

Figure

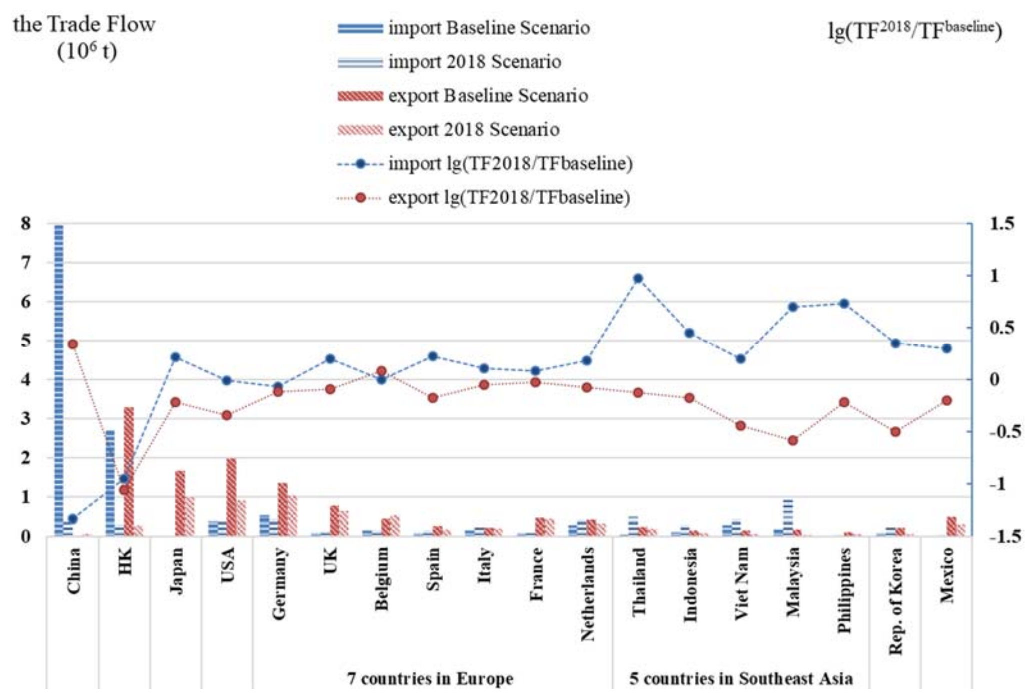

Supplementary Figure 1. The changes of import and export flows after the ban.

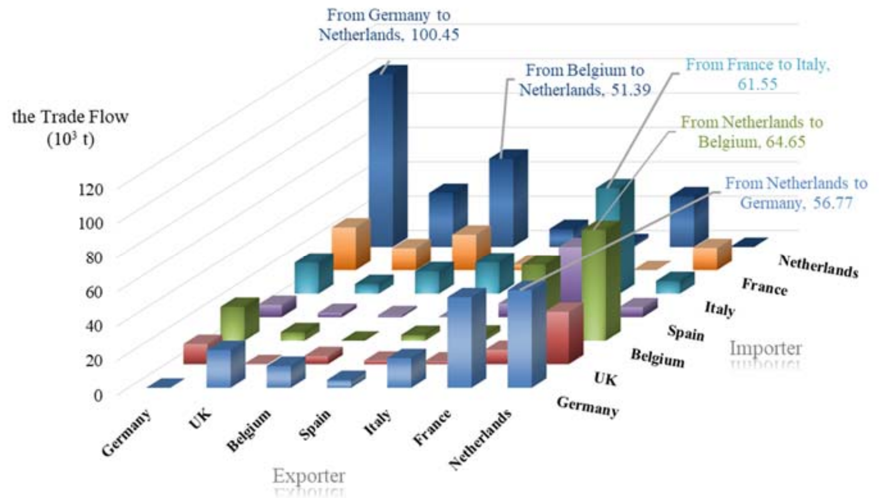

Supplementary Figure 2. The trade flows among Europe 7 members in Baseline Scenario.

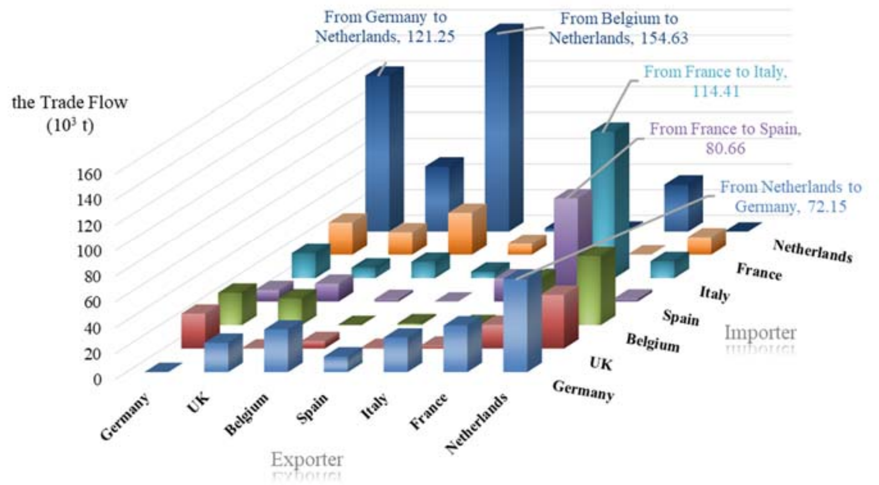

Supplementary Figure 3. The trade flows among Europe 7 members in 2018 Scenario.

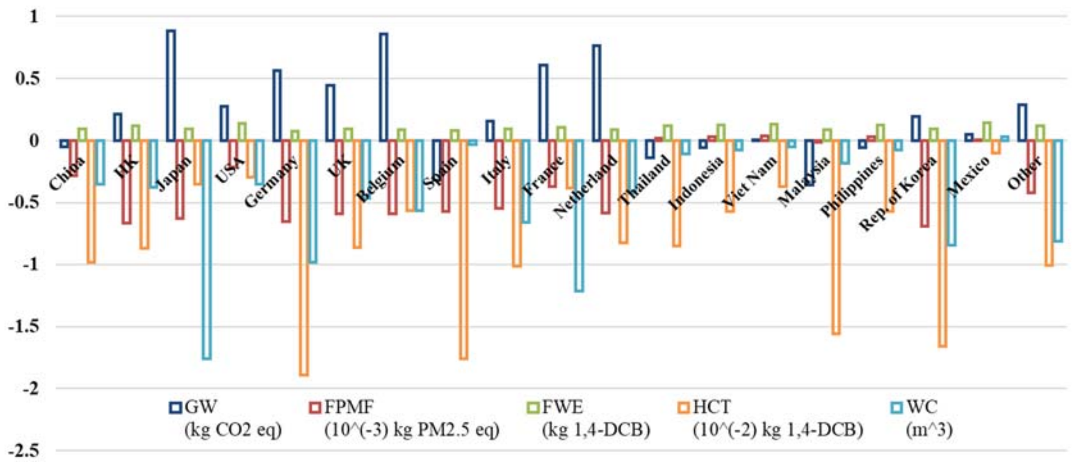

Supplementary Figure 4. The EIT of treating 1 kg of plastic waste in domestic management in 2018.

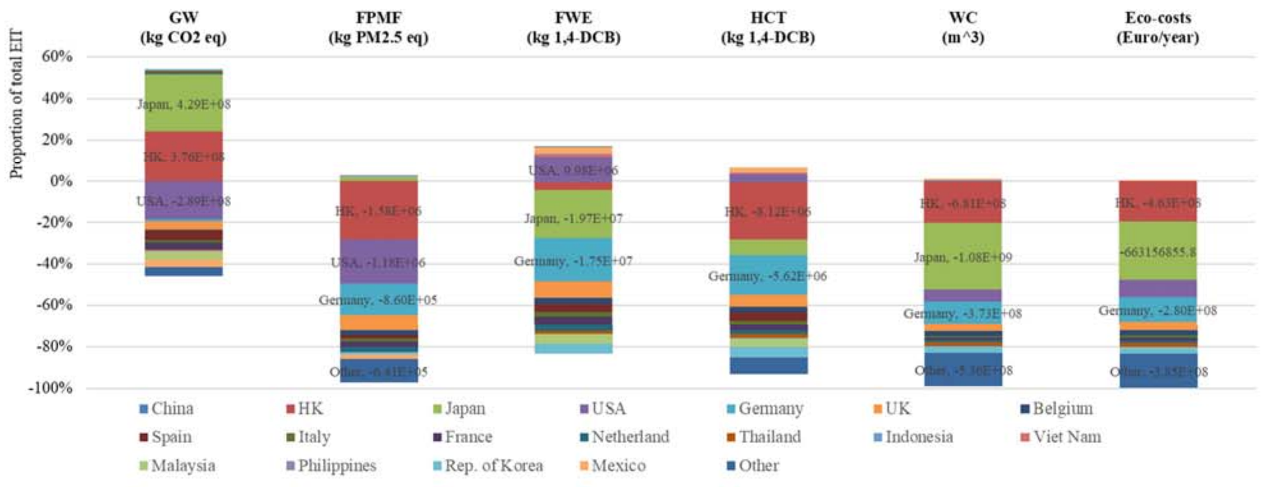

(a) Export countries

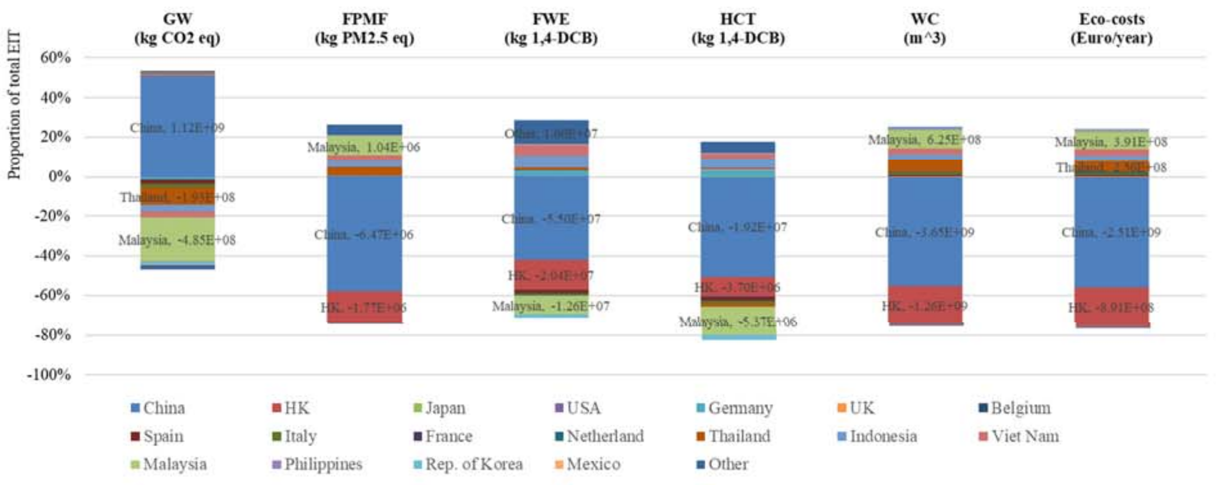

(b) Import countries

Supplementary Figure 5. Contribution of changes of export and import countries to total EIT in 2018 Scenario.

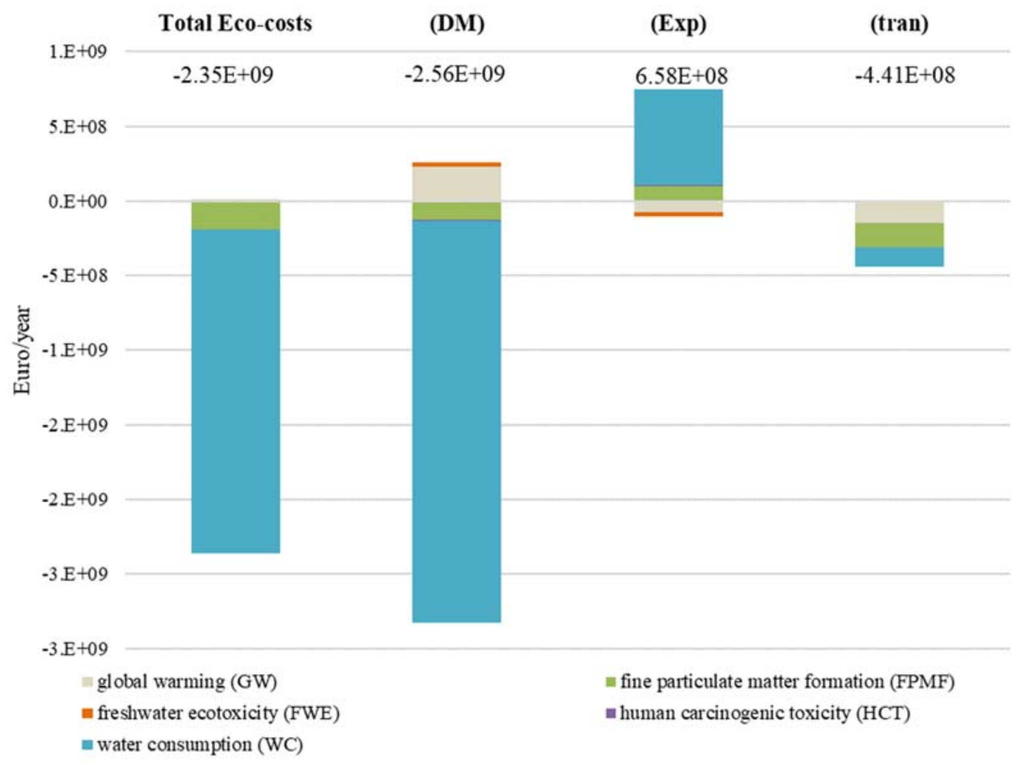

Supplementary Figure 6. Contributions of Eco-costs in 2018 Scenario of each midpoint category.

Supplementary References

1 Chen, Y. *et al.* Life cycle assessment of end-of-life treatments of waste plastics in China. *Resources Conservation and Recycling* **146**, 348-357, doi:10.1016/j.resconrec.2019.03.011 (2019).

2 Recycling of mixed plastics GLO. *Ecoinvent & PRé*.

3 Ritchie, H. & Roser, M. *Plastic Pollution*, <https://ourworldindata.org/plastic-pollution> (2018).

4 The Japan Plastics Industry Federation. *Production of Plastics Materials 2018 Japan*, <http://www.jpif.gr.jp/english/statistics/monthly/2018/2018_production_materials_e.htm> (2019).

5 Inoue, Y. *Japan's Resource Circulation Policy for Plastics*, <http://ec.europa.eu/environment/international_issues/pdf/S2-02-Yusuke%20Inoue.pdf> (2018).

6 Chen, X. The reason and influence of "Plastic resource recycling Strategy" formulated by Japan. *Japanese research* **33**, 29-41 (2019).

7 US EPA. Municipal Solid Waste in the United States: 2009 Facts and Figures, <https://archive.epa.gov/epawaste/nonhaz/municipal/web/pdf/msw2009rpt.pdf> (2010).

8 *Plastics: Material-Specific Data*, <https://www.epa.gov/facts-and-figures-about-materials-waste-and-recycling/plastics-material-specific-data> (2018).

9 Agency, U. S. E. P. Advancing Sustainable Materials Management: 2017 Fact Sheet, <https://www.epa.gov/sites/production/files/2019-11/documents/2017_facts_and_figures_fact_sheet_final.pdf> (2019).

10 PlasticsEurope. Plastics- the Facts 2018. An analysis of European plastics production, Demand and waste data, <https://www.plasticseurope.org/application/files/6315/4510/9658/Plastics_the_facts_2018_AF_web.pdf> (2019).

11 PlasticsEurope. Plastics - the Facts 2017. an Analysis of European Plastics Production, Demand and Waste Data, <https://www.plasticseurope.org/application/files/1715/2111/1527/Plastics_the_facts_2017_FINAL_for_website.pdf> (2018).

12 PlasticsEurope. Plastics - the Facts 2019. an Analysis of European Plastics Production, Demand and Waste Data, <https://www.plasticseurope.org/download_file/force/3183/181> (2020).

13 Bureecam, C., Chaisomphob, T. & Sungsomboon, P.-Y. MATERIAL FLOWS ANALYSIS OF PLASTIC IN THAILAND. *Thermal Science* **22**, 2379-2388, doi:10.2298/tsci160525005b (2018).

14 Jambeck, J. R. *et al.* Plastic waste inputs from land into the ocean. *Science* **347**, 768-771, doi:10.1126/science.1260352 (2015).

15 Ecorea. Environmental Review 2015, Korea. (2015).

16 OECD. Environment at a Glance 2015, <https://www.oecd-ilibrary.org/docserver/9789264235199-14-en.pdf?expires=1558966649&id=id&accname=ocid195144&checksum=77371589C72D2919DC3E6C2EA4062FAB> (2015).
