# Supplementary material for: China’s plastic import ban increases prospects of environmental impact mitigation of plastic waste trade flow worldwide
Source: Nat Commun. 2021 Jan 18;12:425. doi: 10.1038/s41467-020-20741-9 (PMC7813828; doi:10.1038/s41467-020-20741-9)
Supplement: Supplementary file 3 — Reporting Summary [file 41467_2020_20741_MOESM3_ESM.pdf]

nature research

Corresponding author(s): Zongguo Wen

Last updated by author(s): Oct 31, 2020

Reporting Summary

Nature Research wishes to improve the reproducibility of the work that we publish. This form provides structure for consistency and transparency in reporting. For further information on Nature Research policies, see Authors & Referees and the Editorial Policy Checklist .

Statistics

For all statistical analyses, confirm that the following items are present in the figure legend, table legend, main text, or Methods section.

n/a	Confirmed	
☐	☒	The exact sample size (*n*) for each experimental group/condition, given as a discrete number and unit of measurement
☒	☐	A statement on whether measurements were taken from distinct samples or whether the same sample was measured repeatedly
☐	☒	The statistical test(s) used AND whether they are one- or two-sided<br>*Only common tests should be described solely by name; describe more complex techniques in the Methods section.*
☒	☐	A description of all covariates tested
☐	☒	A description of any assumptions or corrections, such as tests of normality and adjustment for multiple comparisons
☐	☒	A full description of the statistical parameters including central tendency (e.g. means) or other basic estimates (e.g. regression coefficient) AND variation (e.g. standard deviation) or associated estimates of uncertainty (e.g. confidence intervals)
☐	☒	For null hypothesis testing, the test statistic (e.g. *F*, *t*, *r*) with confidence intervals, effect sizes, degrees of freedom and *P* value noted<br>*Give P values as exact values whenever suitable.*
☒	☐	For Bayesian analysis, information on the choice of priors and Markov chain Monte Carlo settings
☒	☐	For hierarchical and complex designs, identification of the appropriate level for tests and full reporting of outcomes
☒	☐	Estimates of effect sizes (e.g. Cohen's *d*, Pearson's *r*), indicating how they were calculated

Our web collection on statistics for biologists contains articles on many of the points above.

Software and code

Policy information about availability of computer code

Data collection	1. Most trade data were harnessed from The United Nations Commodity Trade Statistics Database.<br>2. The distance of ocean freighter transport was obtained from https://sea-distances.org.<br>3. All the data of environmental impacts was obtained from SimaPro 8.5.2.
Data analysis	1. Figure 1 is drawn by e!Sankey 4.<br>2. Correlation analysis and Pearson coefficient calculation are conducted by IBM SPSS Statistics 23.0.<br>3. Other statistical analysis is conducted by Excel 2016.

For manuscripts utilizing custom algorithms or software that are central to the research but not yet described in published literature, software must be made available to editors/reviewers. We strongly encourage code deposition in a community repository (e.g. GitHub). See the Nature Research guidelines for submitting code & software for further information.

Data

Policy information about availability of data

All manuscripts must include a data availability statement. This statement should provide the following information, where applicable:
- Accession codes, unique identifiers, or web links for publicly available datasets
- A list of figures that have associated raw data
- A description of any restrictions on data availability

The datasets generated during and/or analysed during the current study are available from the corresponding author on reasonable request.

Field-specific reporting

Please select the one below that is the best fit for your research. If you are not sure, read the appropriate sections before making your selection.

☐ Life sciences ☐ Behavioural & social sciences ☒ Ecological, evolutionary & environmental sciences

For a reference copy of the document with all sections, see nature.com/documents/nr-reporting-summary-flat.pdf

Ecological, evolutionary & environmental sciences study design

All studies must disclose on these points even when the disclosure is negative.

Study description	This study first identified the de facto changes in global plastic waste flow patterns in the aftermath of the ban. Then we further evaluated the de facto environmental impacts and the corresponding Eco-cost of these changes in global plastic waste flow patterns through Life Cycle Assessment (LCA), in which six types of plastics and five midpoint indicators were considered. Last but not least, we adopted Scenario Analysis (SA) to develop two types of prediction scenarios and explore the long-term environmental ramifications in the future.
Research sample	1. Sample countries included China, "17 countries" (Hong Kong, Japan, the United States, seven countries in Europe (Germany, the United Kingdom, Belgium, Spain, Italy, France, and Netherlands), five countries in Southeast Asia (Thailand, Indonesia, Vietnam, Malaysia, and Philippines), Republic of Korea, and Mexico), as well as "other countries".<br>2. Five midpoint indicators in ReCiPe are global warming (GW) (kg CO2 eq), fine particulate matter formation (FPMF) (kg PM2.5 eq), freshwater ecotoxicity (FWE) (kg 1,4-DCB eq), human carcinogenic toxicity (HCT) (kg 1,4-DCB eq) and water consumption (WC) (m3).
Sampling strategy	1. To seek the impacts of the China ban, sample countries included China, "17 countries" which accounted for more than 92% of total plastic waste imports to China in the Baseline, and other countries as a group.<br>2. Among the 18 midpoint indicators provided by ReCiPe, five of them which is relative to the treatment and transportation of plastic waste to a large extent were chosen for analysis.
Data collection	Most trade data were harnessed from The United Nations Commodity Trade Statistics Database, which includes detailed import and export trade data reported by governments of nearly 200 countries and regions. The distance of ocean freighter transport was obtained from https://sea-distances.org. All the data of environmental impacts was obtained from SimaPro 8.5.2, an advanced LCA software developed by PRé sustainability.
Timing and spatial scale	In the Baseline Scenario, historical data of 9 years from 2008 to 2016 were used to describe the general scenario before the ban was issued. As for the data of the 2018 Scenario, we mainly adopted the reporting data in 2018 from Comtrade. Due to the delay in reporting the latest year data by individual countries, some missing data was estimated on the basis of monthly data, mirror data, and others.
Data exclusions	No data were excluded.
Reproducibility	All attempts to repeat the experiment were successful.
Randomization	Not relevant, because it is a research based on factual data on a national scale.
Blinding	Not relevant, because there are no interviewee.

Did the study involve field work? ☐ Yes ☒ No

Reporting for specific materials, systems and methods

We require information from authors about some types of materials, experimental systems and methods used in many studies. Here, indicate whether each material, system or method listed is relevant to your study. If you are not sure if a list item applies to your research, read the appropriate section before selecting a response.

Materials & experimental systems

n/a	Involved in the study	
☒	☐	Antibodies
☒	☐	Eukaryotic cell lines
☒	☐	Palaeontology
☒	☐	Animals and other organisms
☒	☐	Human research participants
☒	☐	Clinical data

Methods

n/a	Involved in the study	
☒	☐	ChIP-seq
☒	☐	Flow cytometry
☒	☐	MRI-based neuroimaging
