# Supplementary material for: China’s plastic import ban increases prospects of environmental impact mitigation of plastic waste trade flow worldwide
Source: Nat Commun. 2021 Jan 18;12:425. doi: 10.1038/s41467-020-20741-9 (PMC7813828; doi:10.1038/s41467-020-20741-9)
Supplement: Supplementary file 4 — Source Data [file 41467_2020_20741_MOESM4_ESM.zip › 4-Source data/Source Data- Figure 3.pptx]

## Slide 1
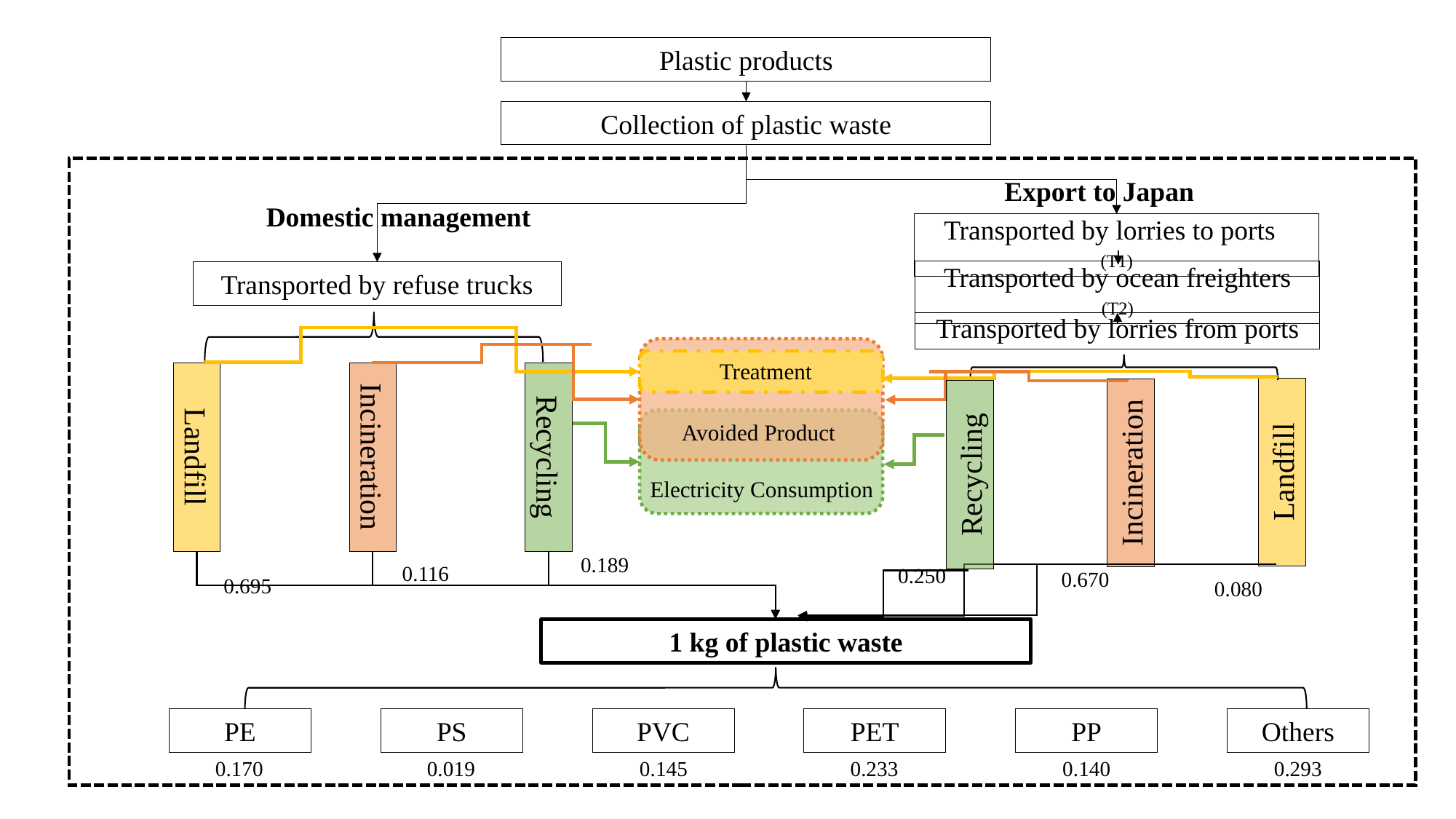

Plastic products
Collection of plastic waste
Export to Japan
Domestic management
Transported by lorries to ports (T1)
Transported by ocean freighters (T2)
Transported by refuse trucks
Transported by lorries from ports
Treatment
Avoided Product
Electricity Consumption
Recycling
Landfill
Incineration
Landfill
Incineration
Recycling
0.189
0.116
0.250
0.670
0.695
0.080
1 kg of plastic waste
Others
PET
PP
PE
PS
PVC
0.170
0.019
0.145
0.233
0.140
0.293
