# Supplementary material for: China’s plastic import ban increases prospects of environmental impact mitigation of plastic waste trade flow worldwide
Source: Nat Commun. 2021 Jan 18;12:425. doi: 10.1038/s41467-020-20741-9 (PMC7813828; doi:10.1038/s41467-020-20741-9)
Supplement: Supplementary file 4 — Source Data [file 41467_2020_20741_MOESM4_ESM.zip › 4-Source data/Source data- Figure 1.pptx]

## Slide 1
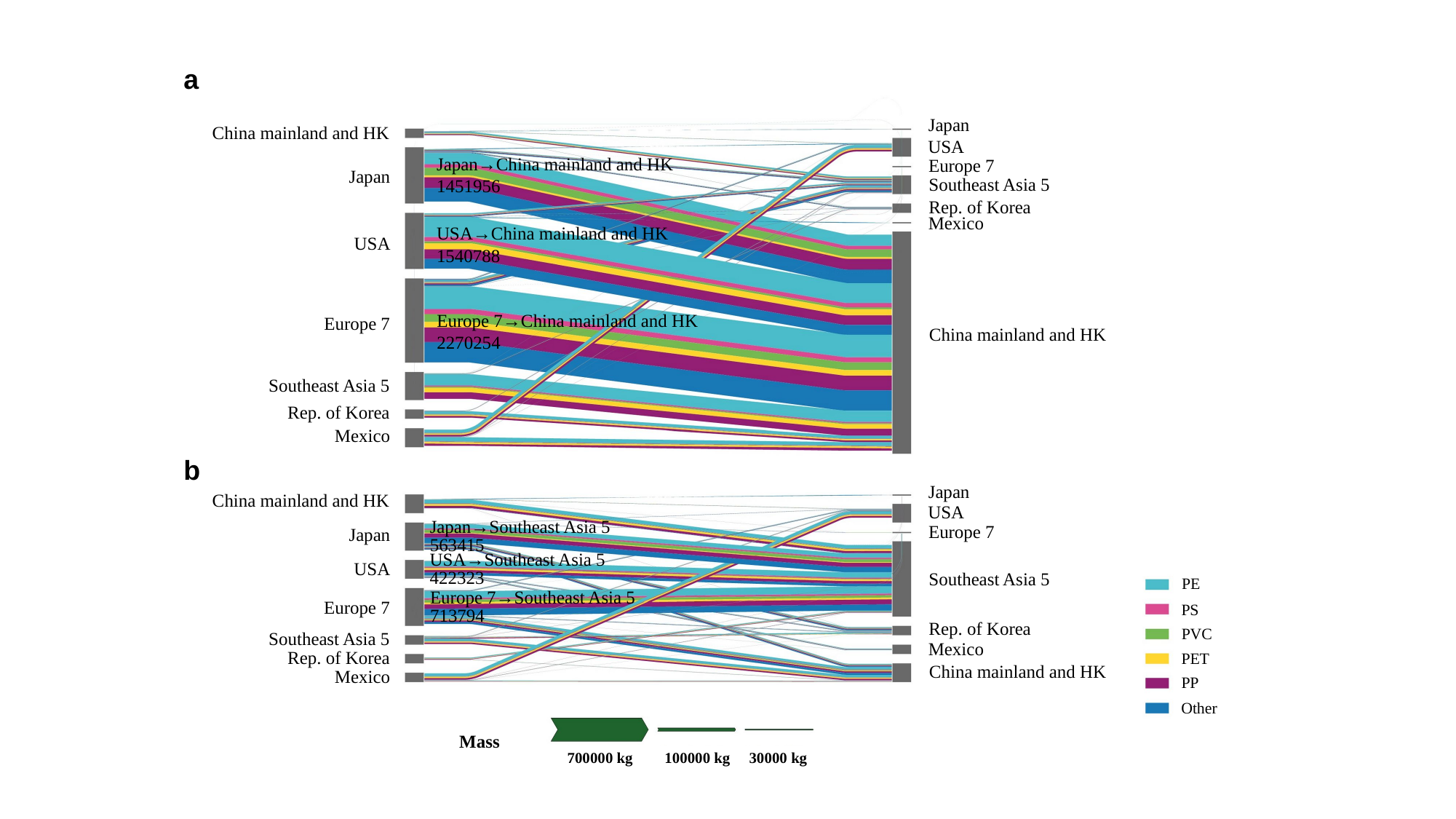

a
Japan
China mainland and HK
USA
Japan→China mainland and HK
1451956
Europe 7
Japan
Southeast Asia 5
Rep. of Korea
Mexico
USA→China mainland and HK
1540788
USA
Europe 7→China mainland and HK
2270254
Europe 7
China mainland and HK
Southeast Asia 5
Rep. of Korea
Mexico
b
Japan
China mainland and HK
USA
Japan→Southeast Asia 5
563415
Europe 7
Japan
USA→Southeast Asia 5
422323
USA
Southeast Asia 5
Europe 7→Southeast Asia 5
713794
Europe 7
Rep. of Korea
Southeast Asia 5
Mexico
Rep. of Korea
China mainland and HK
Mexico
PE
PS
PVC
PET
PP
Other
Mass
700000 kg
100000 kg
30000 kg
