# Supplementary figures and images for: China’s plastic import ban increases prospects of environmental impact mitigation of plastic waste trade flow worldwide
Source: Nat Commun. 2021 Jan 18;12:425. doi: 10.1038/s41467-020-20741-9 (PMC7813828; doi:10.1038/s41467-020-20741-9)

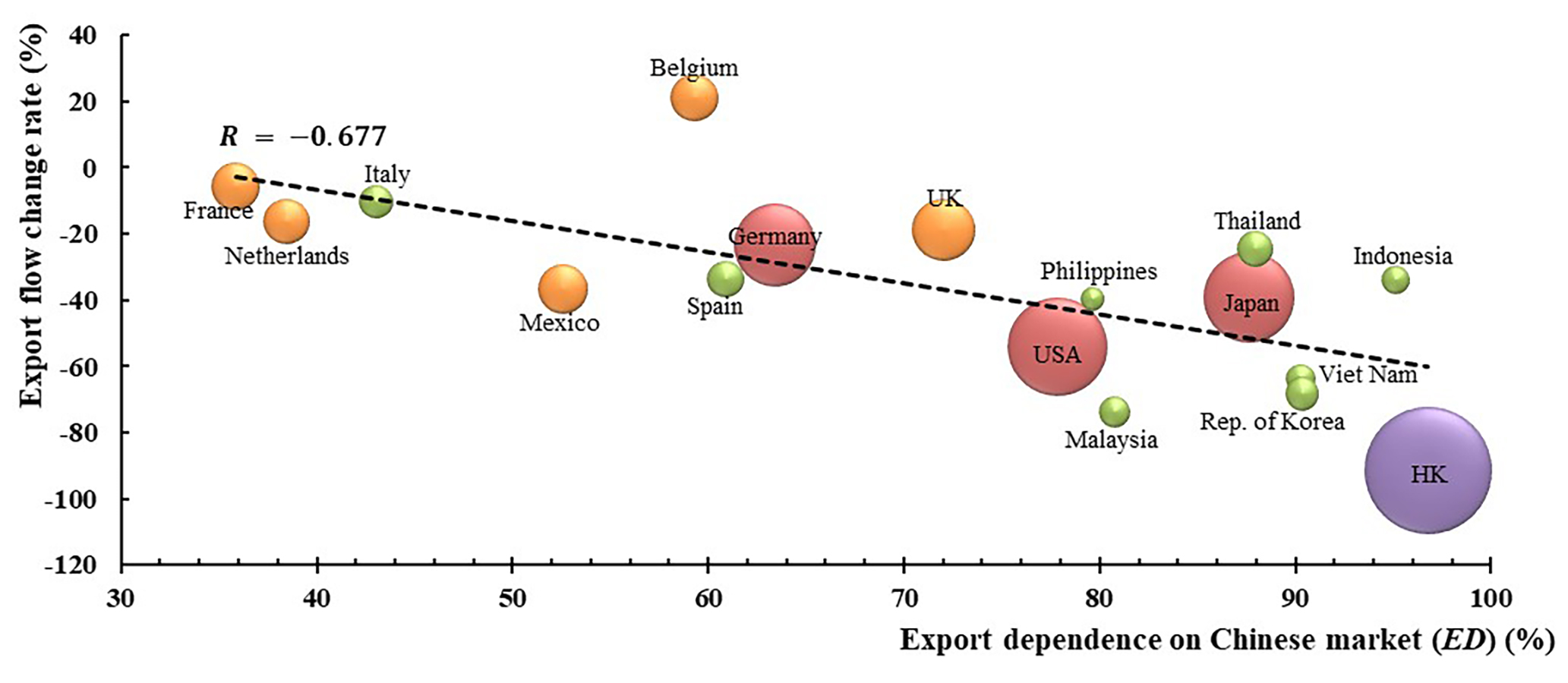

Supplement: Supplementary file 4 — Source Data [file 41467_2020_20741_MOESM4_ESM.zip › 4-Source data/Source data- Figure 2.jpg]

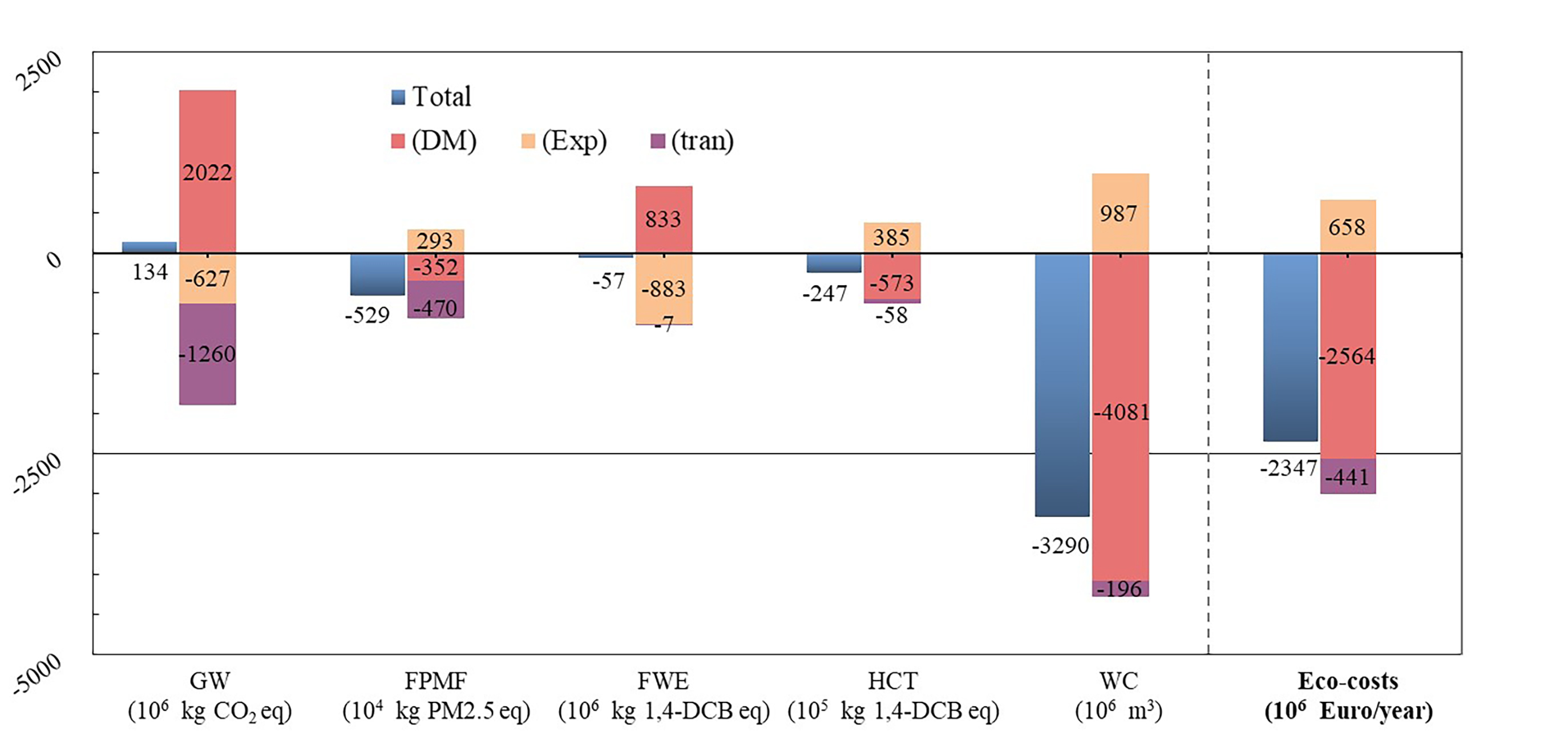

Supplement: Supplementary file 4 — Source Data [file 41467_2020_20741_MOESM4_ESM.zip › 4-Source data/Source data- Figure 4.jpg]

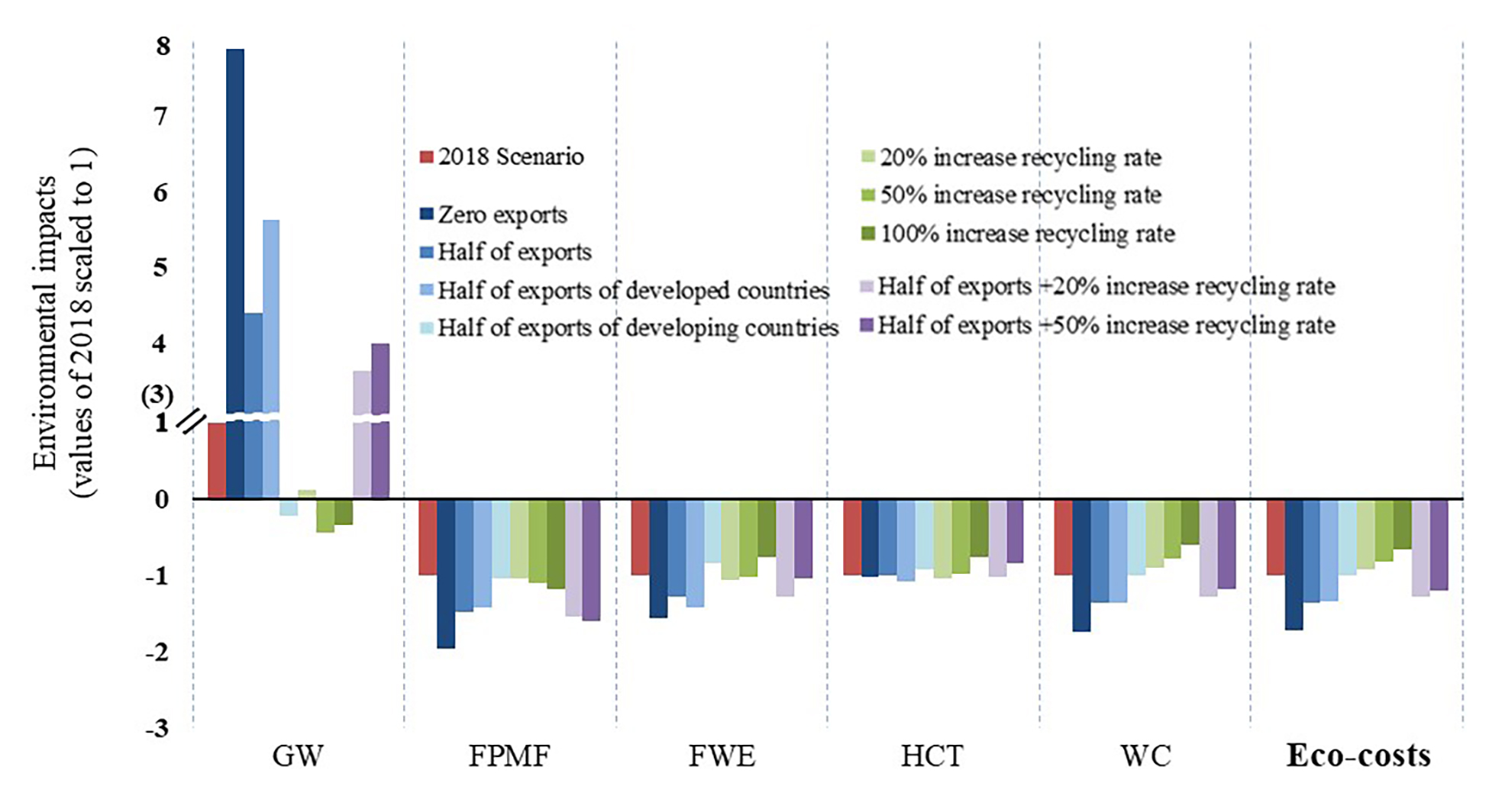

Supplement: Supplementary file 4 — Source Data [file 41467_2020_20741_MOESM4_ESM.zip › 4-Source data/Source data- Figure 5.jpg]
